# Children’s Narrative Elaboration After Reading a Storybook Versus Viewing a Video

**DOI:** 10.3389/fpsyg.2020.569891

**Published:** 2020-10-16

**Authors:** Camilla E. Crawshaw, Friederike Kern, Ulrich Mertens, Katharina J. Rohlfing

**Affiliations:** ^1^Psycholinguistics, Faculty of Arts and Humanities, Paderborn University, Paderborn, Germany; ^2^Language and Communication, Faculty of Rehabilitation Sciences, TU Dortmund University, Dortmund, Germany; ^3^Faculty of Linguistics and Literature, Bielefeld University, Bielefeld, Germany

**Keywords:** narrative skill development, narrative retelling, narrative elaboration, digital media, non-verbal IQ

## Abstract

Previous studies have found that narrative input conveyed through different media influences the structure and content of children’s narrative retellings. Visual, televised narratives appear to elicit richer and more detailed narratives than traditional, orally transmitted storybook media. To extend this prior work and drawing from research on narrative elaboration, the current study’s main goal was to identify the core plot component differences (the *who*, *what*, *where*, *when*, *why*, and *how* of a story) between children’s retellings of televised versus traditional storybook narratives. However, because children also differ individually in their IQ, we further incorporated this variable into our analysis of children’s narrative retellings. For our purpose, a novel coding schema was developed, following and extending the existing narrative elaboration approaches. Participants were 46 typically developing children aged 4–5 years from Germany. The current study incorporated two narrative input conditions to which children were randomly assigned: in the video condition, children watched a non-verbal, visually conveyed, televised story from a DVD; and in the book condition, children read the story with an adult and experienced an orally conveyed version in the form of a book with minimal accompanying pictures. In both conditions, the same story was conveyed. After including IQ as a covariate in our analyses, results show that the children from the video condition gave significantly more elaborated retellings, particularly across the *who*, *what*, and *where* (sub-)components. Differences between the conditions in the component *when*, *how* and *why* did not reach statistical significance. Our findings indicate that different media types entail differential cognitive processing demands of a story, resulting in type-specific memories and narratives. The effect of different medial conditions was significant and persisted when individual differences in cognitive development were considered. Consequences for children’s development, education, and interaction with and within today’s digital world are discussed.

## Introduction and Prior Work

The literature on children’s development of narrative skills is both vast in volume and broad in focus. In this paper, preschool children’s narrative elaborations are investigated in relation to medial input and their cognitive development. This focus takes into account the structural and social contexts underlying the components of a narrative, considering and drawing from wide-ranging research traditions with diverse theoretical underpinnings.

### Narrative Structure and Narrative Elaboration

A story’s plot components occur within a structural narrative context. In oral narratives, past events are retold and evaluated from the speaker’s perspective, structured into chronological and causal sequences of sub-events and reproduced with linguistic and multimodal resources (Burdelski and Evaldsson, 2019; Heller, 2019; Makdissi et al., 2019; Takagi, 2019). For this purpose, storytellers need to leave the here and now of an interaction (Bühler, 1934/2011) and create a fictional world in which the narrated events occur, allowing them to narrate events about persons displaced from the here (space) and now (time), packaging these concepts accessibly for their listeners (Heller, 2019; Nicolopoulou, 2019; Takada and Kawashima, 2019). In doing so, they establish a situation of “joint imagination” in which the hearer is also a key contributor to the storytelling process (Heller, 2019, p. 168). Many studies have shown that narrative structure and content is not the product of the speaker alone but co-constructed and jointly achieved in the process of storytelling (e.g. Mandelbaum, 2012). While the ability to create a story with consideration of what the hearer knows and can imagine is crucial to tell a good story, here we focus on the components that a story comprises. These represent various types of information about a narrative event, which the teller can package and structure into an elaborated and entertaining story for their listener. According to Mandler and Johnson (1977, p. 111), narrative components can be summarised into a “story schema,” i.e. an idealised representation of a typical story. In this sense, structure is a transferrable aspect of narratives and while it is crucial to the production of narratives, so too is the unique story and plot content delivered within the structural elements. Children start with limited linguistic means with which they can express the elements but over time the “expressive options” increase and “come to fulfil more specific and differentiated functions” (Veneziano and Nicolopoulou, 2019, p. 3). In this way, *narrative elaboration* involves the integration of structure and content within the process of telling a story and effectively communicating its events.

Linguistic research has now shown that narratives feature components produced systematically and in a specific order during the process of storytelling (see Veneziano and Nicolopoulou, 2019 for a recent summary). These include: (a) an orientation providing “relevant setting information” (Polanyi, 1985, p. 191) about the situation and the people involved, thus locating the event told in space and time, (b) a complication action, and (c) an evaluation section that can include personal, emotional and evaluative comments. Finally, (d) a coda takes the setting back to the present. The complicating action constitutes the point of the story (Polanyi, 1985), or its “high-point” (Peterson and McCabe, 1983, p. 37), and bears important functions for the “reportability” or “tellability” (Norrick, 2004, p. 86) of a story. This is supported by the referential and temporal “connectivity” of a story (Veneziano and Nicolopoulou, 2019). Labov and Waletzky (1967, p. 34–35) proposed that evaluation tends to cluster around emotional “high points” (during the complicating action component). Evaluation is important for achieving tellability and usually necessitates inferencing, because it provides explanations of why events occur, in particular the actions of characters in the story, and involves reference to feelings, thoughts and intentions (Eaton et al., 1999). Inferencing can, however, also be required for non-evaluative story-sequencing and linguistic details (e.g. anaphoric pronouns). Conversely, sequencing could also be viewed as implicitly supporting inferencing, functioning as a precursor to more explicitly expressed causality and consequences.

In the area of language acquisition, Saywitz and Snyder (1996) developed the *Narrative Elaboration* (NE) procedure to elicit and promote children’s recall of narrative events (framed in our work as plot components), particularly for use within forensic environments. The design of this procedure was also based on work on story grammars as well as work on script theories and event knowledge (Flavell, 1970; Stein and Glenn, 1978; Nelson, 1986), separating out a narrative into “logically salient” categories in order to help children conceptualise the parts of a story and guide their event recall (Saywitz and Snyder, 1996, p. 1348; Saywitz et al., 1996). These categories represented various plot components and consisted of: participants, setting, actions, conversation/affective state, corresponding to questions about the *who*, *what*, *where*, *when*, *how*, and *why* of a story (Saywitz et al., 1996; Camparo et al., 2001; Brown and Pipe, 2003a). Various studies (Saywitz et al., 1996; Camparo et al., 2001; Brown and Pipe, 2003a,b) have since examined the effectiveness of this elicitation technique on the accuracy of children’s free and cued event recall.

A story’s components, corresponding to details concerning the *who*, *what*, *where*, *when*, *why*, and *how* of its events (Saywitz and Snyder, 1996; Saywitz et al., 1996), can be perceived as the “content” of a story, but these elements are also focused on and feature within the different structural elements to varying extents. The *who*, *where* and *when* represent setting information (Norbury and Bishop, 2003) and thus in conjunction with the *what* could be seen as representing aspects of orientation (who did what, as well as where and when they did it). The *how* could feature both as a visually more descriptive element (how something looked or was done) as well as a method for providing affective information and details about the characters (how they felt), straddling the elements of orientation, complicating action or high point, and informing the evaluation of the story. The *why*, then, represents the evaluative and goal-oriented elements of the story. It draws on sequencing to provide information about causality and consequences, incorporates affective stance, and thus does important work in establishing a story’s reportability. In the sense that a speaker creates a fictional world to tell a story (s.a.), the *who* also relates to bodily displacement, the *where* to spatial displacement, and its *when* to temporal displacement. In studying the emerging elaboration of children’s narratives, it is thus difficult to decouple the aspect of a complete narrative structure being provided from the elaboration itself. Both aspects are often intertwined in the literature.

### Narrative Development

Combining plot components to form a whole story draws on various abilities. Becoming able to knit narrative content into an accessible structure is driven by children’s linguistic and cognitive development as well as influenced by social and situational factors. This development occurs over a protracted period from the production of early, simplistic narratives at 2 years of age to far more complex narratives at 10 years of age, continuing to mature even into their adolescence (Stadler and Ward, 2005; Quasthoff et al., 2017; Heller, 2019; Theobald, 2019). When constructing a story, young children struggle to effectively incorporate important details about the *who*, *what*, *where*, *when*, *why*, and *how* of a story (Saywitz and Snyder, 1996; Saywitz et al., 1996). The reason for this appears to be manifold: It may be due to limited event knowledge or understanding of causal and temporal relations and still-developing linguistic (i.e., grammatical and lexical) skills (Colletta et al., 2010; Hamilton et al., 2020). In this respect, the ability to express temporal and causal sequencing in a more differentiated way depends on the complexity of linguistic means available to the speaker (Veneziano and Nicolopoulou, 2019). The reasons for children’s struggle with incorporating important details can also be related to limited discursive and sociocognitive skills which enable them to effectively orient their listener and adjust the narrative to their informational needs (Saywitz and Snyder, 1996; Saywitz et al., 1996; Genereux and McKeough, 2007; Colletta et al., 2010; Melzi et al., 2011; Pavias et al., 2016; Dore et al., 2018; Hamilton et al., 2020).

Whereas the above literature demonstrates the linguistic variability in the acquisition of narrative structure and content, the explanations of this variability are also associated with cognitive and social influences. Little is known about the influence of IQ on narrative skills and elaboration. Children will naturally approach the telling of a narrative pre-furnished with varying individual assets or levels of ability and some researchers have touched upon this idea in their work with children with learning disabilities (Humphries et al., 2004; Stetter and Hughes, 2010; Shamir et al., 2018). In order to retell a story, a child must make inferences about and remember information about the original story’s plot. To linguistically construe an event (Nicolopoulou, 2019), a child must retain or conceive of relevant vocabulary and grammatical structures that appeared in or pertain to the original story (Humphries et al., 2004; Shamir et al., 2018) without relying on implicitly shared knowledge. Because of this challenge, studies report that children’s early narratives are initially heavily dependent on scaffolding activities from more competent speakers before they develop strategies for recalling and providing more elaborate details about a story as well as producing a coherent and contextualised discourse unit without adult support (Saywitz and Snyder, 1996; Saywitz et al., 1996; Kern and Quasthoff, 2005; Haden et al., 2009; Melzi et al., 2011; Quasthoff et al., 2017; Theobald, 2019). They also rely on scaffolding before they become able to establish reportability for their audience (Kern and Quasthoff, 2005). Families are thus seen as the primary context within which children’s storytelling skills emerge and evolve (Hyvärinen, 2008; Heller, 2019; Takada and Kawashima, 2019; Takagi, 2019).

Because of the variability of the linguistic structure and content that is associated with these influences, methodologically, it is a challenge to assess children’s narrative performance properly. Prior works have taken differing yet overlapping approaches to the delineation and classification of children’s narratives. Following Labov (1972) and McCabe and Peterson (1991) devised the method of high point analysis in order to examine children’s narrative macrostructure across a developmental continuum, identifying seven progressive steps of narrative structure (also: Peterson and McCabe, 1983). Building from Stein and Glenn’s (1979) work on story grammar, Stadler and Ward (2005) took a similar approach in developing their model for narrative development which included the following levels: labelling, listing, connecting, sequencing, and narrating. Their approach lacked this socially interactive concept of a “high point” but, as discussed above, sequencing is also an important aspect of tellability and supports inferencing. Taking a somewhat different approach by extending the concept of plot components derived from the (1967) work of Labov and Waletzky (1967), Kemper (1984) proposed that children first acquire an inventory of diverse plot components, then the rules for coordinating them, and finally the rules for embedding these components recursively, with stories conforming to grammatical principles governing structural components and their organisation. More recently, Makdissi et al., 2019, p. 51) introduced a narrative recall coding scale that offers a hierarchy starting from naming objects, recollection of isolated actions and developing further to temporal, causal structuring and finally explanations. However, these studies all focused on children’s narrative content primarily within the context of structure, and while structure is an important aspect, the varying content and the unique plot components that children choose to incorporate within a transferrable structural schema can also contribute to our understanding of their narrative development. This is an area that should be further addressed in the literature.

Another aspect challenging the methodology in assessing children’s narrative performance is linked to the material that is supposed to elicit children’s narration. McCabe and Rollins (1994) drew attention to the manifold issues involved in eliciting narratives from children. Studies comparing narrative retellings (e.g. of fictional stories and personal experiences) to narrative generation from picture stimuli have shown that elicited narratives based on pictures taken out of context barely reach the quality of situated personal narratives, and that retold narratives appear to be longer and more detailed with more frequently complete episodes (Liles et al., 1989; Merritt and Liles, 1989). This has consequences for the validity and generalisability of research findings. Differences in children’s performance might also result from the interactive process of constructing a narrative for and with an audience. This is relevant to children’s experience of both real-life and experimental settings. Certain types of stimuli such as televised media or storybooks appear to be much more effective at stimulating and scaffolding children’s production of narratives, although it is as yet still unknown which forms of media generate comparatively greater outcomes. In accordance with our aim to explore the influence of different media on narrative elaboration, we present further related research in the following section.

### Media Effects on Narrative Elaboration

Given that children today are growing up in a digital world, it is important to address questions concerning the impact digital media might have on cultural traditions such as storytelling and how children engage with them. In particular, the ways in which the content and structure of different input medias or experiences might bias or influence narrators’ strategies. This might include which narrative details they form stronger mental representations of or consider most pragmatically salient or appropriate for retelling to their listeners. Differential opportunities to access print media exist across the socio-economic spectrum but the majority of households in developed countries have access to televisions and televised narratives (Linebarger and Piotrowski, 2009; McPake et al., 2013). The benefits of television in comparison to traditional media had previously been obscured, but more recently it has been shown that televised narratives (among other digital media or enhancements) can actually have positive impacts on children’s development in different ways (Krendl and Watkins, 1983; McPake et al., 2013; Sarı et al., 2019). Yet, few studies have examined its impact on children’s narrative development and production, let alone in comparison to traditional static or storybook media, and those that have done so tend to focus on either cognitive processing (e.g. Krendl and Watkins, 1983), story comprehension (e.g. Beentjes and van der Voort, 1991a,b; Podszebka et al., 1998; Linebarger and Piotrowski, 2009) or word learning (e.g. Podszebka et al., 1998; Diehm et al., 2020).

The use of video narratives in experiments has been observed to lead to the production of richer and more detailed narratives: Processing and encoding them may be cognitively easier for children, given that they tend to fall back onto reporting information presented in the visual format (Beck and Clarke-Stewart, 1998; Eaton et al., 1999; Linebarger and Piotrowski, 2009; Diehm et al., 2020). In a (1983) study by Krendl and Watkins, results indicated that viewers engaged in an active and differential processing of televised information, consequently acquiring a stronger mental encoding, a more sophisticated understanding, and better recall of the material. They argued that people have a lesser degree of control over the pace of its presentation in contrast to book reading and thus may activate different cognitive methods for processing the information conveyed, potentially at different levels of meaning. In addition to this, viewers of all ages need to continually revise their hypotheses about a televised narrative’s implicit plot and sub-plots, and this uncertainty may result in increased levels of attention and cognitive effort (Krendl and Watkins, 1983). A better comprehension of the original material would certainly support children’s ability to successfully retell a story and if children employ different strategies when processing narrative input, this could shape their retellings. From the findings of their (1998) study with 66 5-years-old, Beck and Clarke-Stewart also proposed that television could be especially effective at presenting stories (facilitating greater narrative elaboration) because (a) it is enjoyable and maintains children’s attention, (b) information is often redundant (allowing for children to be momentarily distracted but still acquire the story’s gist), (c) the dual presentation (visual and verbal) of information has a beneficial effect on memory, and (d) audiovisuals can depict affective content more transparently, making it easier to perceive and remember (also: Linebarger and Piotrowski, 2009).

Linebarger and Piotrowski (2009) investigated the effects of viewing different types of televised programmes (expository frameworks, embedded narrative, and traditional narrative, as well as a no viewing condition) on story knowledge and narrative skills in 311 at-risk pre-schoolers, and found that story knowledge scores (the ability to sequence story events and then tell stories around these events) and narrative skills (narrative involvement, retelling, explicit comprehension, and implicit comprehension) were higher in children assigned to either narrative condition. Sarı et al. (2019) investigated the impact of digital enhancements of storybooks on narrative comprehension and word learning. These types of digitally enhanced e-books could be seen as a bridge between traditional print and modern televised media. Their study with 99 children between 4 and 6 years of age covered four experimental conditions: Static illustrations with/without music or sounds, and animated illustrations with/without music or sounds. They found that visual enhancements and film-like story presentation benefited story comprehension. These findings are in line with the previous work, indicating that, overall, televised narratives boost story comprehension in comparison to traditional oral narratives, perhaps as a result of visual information being easier to process than verbal or language-based input.

Despite the ubiquity of television narratives in the everyday lives of many people today, very few studies have been conducted that actually compare the retellings of storybook and video narratives and even fewer have done so with very young children. In a study with four classes of children in the eighth grade (*N* = 70), Podszebka et al. (1998) found that children who read a book version of a story better acquired target vocabulary, while those who viewed a video version better comprehended it. Diehm et al. (2020) recently investigated the effect of the presentation format of a story (static picture book versus animated video) on the language content of preschool children’s narrative retellings), finding that typically developing children demonstrated a higher quantity and quality of language within a story retelling setting after viewing an animated video than after viewing images from the same video presented in a static picture book format. The findings of both Podszebka et al. (1998) and Diehm et al. (2020) also suggest that the content of children’s narratives may be differentially affected by the medium of input to which they are exposed. With regard to story content, Beentjes and van der Voort (1991a;b) conducted two studies comparing children’s written retellings of a printed story versus its video version, the first with 88 children in grades 4–6 and the second with 127 children aged 10–12. They found that the children in the video condition included more scenes (narrative events) in their essays and had fewer errors, while the children in the printed book condition were better at specifically referencing characters and using descriptive details in their retellings (Beentjes and van der Voort, 1991a). They further found that recall of the video and storybook narratives varied with age: the younger children’s recall of the film was more complete than that of the book, although this effect dropped off in the older children (Beentjes and van der Voort, 1991b). Podszebka et al. and Beentjes and van der Voort’s findings support the previously discussed hypotheses of Krendl and Watkins (1983); Beck and Clarke-Stewart (1998), Eaton et al. (1999), and Linebarger and Piotrowski (2009) that televised narratives are more strongly mentally encoded, leading to more detailed retellings.

Taken together, the above research appears to demonstrate that children better encode and recall original story input after watching a televised narrative in contrast to a traditional storybook format, leading to narrative retellings which are more elaborate and detailed. However, this prior work has not focused on the specific ways in which children’s retold narratives differ in terms of story and plot components after viewing a video versus reading a book. The study we report here has attempted to address this gap in the research by exploring how specific plot components of a retold story may be affected by the two conditions. For this purpose, a coding system had to be developed in order to identify core aspects of story content that are linked to and reflect narrative structure.

## The Current Study

Our study worked from a psycholinguistic perspective to examine narrative content and elaboration grounded in language-based categories. Our aims were threefold: The first aim was to develop a functionally operational coding system fit for the purpose of analysing narrative content and elaboration. The second aim was to use this coding system to investigate whether the focus of children’s elaborative narrative content differed between their retellings of two narrative input conditions: a verbal narrative conveyed to the children from an illustrated storybook by a caregiver at home and a non-verbally conveyed narrative in the form of an animated video with sound effects that the children watched at home. Building on the previous research demonstrating that televised narratives are better encoded and thus lead to more detailed retellings, it was hypothesised that the children viewing the video version of the story would produce more elaborated retellings than those who had experienced the traditional storybook version. For the third aim of our study, we followed the literature documenting the influence of cognitive development on narrative retelling success and incorporated children’s scores on a non-verbal intelligence test (IQ) as a covariate within our analyses to address the lack of its inclusion in prior studies.

### Method

The focus of this particular study is on the narrative retelling setting of a wider study on children’s linguistic and gestural development which involved multiple settings (Rohlfing et al., in prep).

### Ethics

The ethical considerations for all procedures, measures, and assessment of participants were evaluated and granted approval by the ethical committee of the Bielefeld University (EUB 2014-111). Parents of the children participating gave informed consent and the children were given the opportunity to withdraw from the experimental interaction at any time.

### Participants

A sample of 55 children between the ages of 4–5 years old were recruited for the wider study. Of these 55 participants, 9 had to be excluded such that the narrative retelling data from 46 participants (27 male and 19 female) could be used for our analyses. Of the 9 excluded from the analyses, 6 children experienced the wrong story at home, 2 children used the book or DVD-cover when retelling the story and 1 caregiver already knew the story. The ages of these 46 children in months at point of testing ranged between 45 and 61 months (*M* = 50; *SD* = 3.4). Data concerning the children’s IQ scores was collected in a follow-up session which 7 participants did not attend, such that it could only be collected in 39 of these 46 cases (see Table 1 for a summary).

**TABLE 1:** Details of the data collected and participant numbers per condition.

**Collected data**	**Condition**	
	Video	Storybook
§4.1 Dimension Analyses	21 (10 male and 11 female)	25 (17 male and 8 female)
§4.2 Medial Condition Analyses	16 (9 male and 7 female)	23 (16 male and 7 female)

### Stimuli

In the book condition, we used a published German translation of a Czechian children’s storybook titled “The mole and the green star” (Doskočilová et al., 1998/2013). This book is commercially available as is the DVD version used in the video condition. This material has the same pictures: Moving pictures for the video condition and selected static pictures for the book condition. In the story, the mole protagonist wakes up from hibernation, begins spring-cleaning his burrow and finds a green gemstone in the process. The mole believes that this is a green star that has fallen from the sky and spends the rest of the book trying to put it back in the sky with help from his friends, including the moon who finally helps him achieve his goal. The plot of the book came from a non-verbally presented cartoon (Miler, 1969), and we used this cartoon in the video condition. The book and video were almost identical in underlying plot with very minor differences in scene emphasis or focus as a result of the mode of presentation.

### Measures

#### IQ

We assessed the children’s IQ using the measure SON-R (Tellegen et al., 2007), which creates a generalised composite measure of children’s intellectual abilities from two sub-tests: SON-H covering spatial thinking skills, and SON-D corresponding to abstract thinking skills.

#### Narrative Condition

The recruited children were randomly assigned to either of two narrative input conditions: the traditional illustrated storybook format or the non-verbal animated cartoon-video format. A strength of this study lies in that both narratives depicted the same underlying events, as they both told the story of “The mole and the green star” (Miler, 1969; Doskočilová et al., 1998/2013), allowing for direct comparison of the content of the children’s narrative retellings between the two conditions.

#### Data Collection

Each child’s narrative retelling was audio- and video-recorded, transcribed, and coded using ELAN (2019). The child retold the narrative to a caregiver who had not been present during the original presentation of the narrative input. The setting was designed to promote a natural narrative retelling interaction between the child and caregiver, and for this reason, neither the child nor the caregiver were instructed to behave or speak in any specific way during the exchange.

## Coding

To compare children’s narratives across two conditions, a novel coding system was developed. It took a qualitative content analysis approach (following Schreier, 2012) extending Saywitz and colleagues’ work on the narrative elaboration technique/procedure of cued event recall (Saywitz and Snyder, 1996; Saywitz et al., 1996). The extension of Saywitz and colleagues’ narrative elaboration categories (participants, setting, actions, conversation/affective states, and consequences) pertains to the underlying question cues about the *who*, *what*, *where*, *when*, *why*, and *how* of the story (see Table 2). Importantly, the categories also reflect narrative structure, as they routinely occur in the various structural parts of a narrative (s.a., section “Introduction and Prior Work”).

**TABLE 2:** Comparison of narrative elaboration categories and coding schema components.

**Saywitz and colleagues’ categories**	**Extension for this study**
Characters	*Who*—characters named directly or indirectly in the story
Setting/Location	*Where and When*
	• *Where* (representing the spatial element)
	• *When* (representing the temporal element)
Actions	*What*—for this component we coded verbs (linguistically encoded actions/states)
Affective States	*How*—extended to code for adjectives as well as adverbs of manner and degree
Consequences	*Why*—causal connectives and purpose/goal-oriented elements

As can be seen in Table 2, six categories (who, what, where, when, why, how) formed our main components for the core and elaborative information of children’s narrative retellings. These were then segregated into three dimensions, loosely following syntactic structure. The main reason behind this was the assumption that syntactic components of a clause match major narrative components on a sentence level, and emerging syntactic complexity reflects increasing narrative complexity, although of course a story is also influenced by wider pragmatic aspects and is more than just the sum of its parts. However, by including linguistic categories that are relevant on a text level as well (such as nouns vs. pronouns, and temporal adverbs), we hope to catch aspects of narrative complexity above the sentence level as well.

Our dimensions included: Dimension 1 (*who*, *what*) reflecting the basic necessary linguistic properties of a sentence required to orient the listener (predicate: Subject and verb); Dimension 2 (*where*, *when*, *how*) representing the inclusion of (slightly more optional) temporal, spatial, and descriptive information; and finally Dimension 3 (*why*) incorporating causality, the most complex element of the stories (see also Makdissi et al., 2019). Following Kemper’s (1984) model of narrative competence, Dimensions 1 and 2 represent the content and diverse plot components of children’s narratives which are first acquired and elaborated on before children can progress to relating them and creating the causal structure of a story within Dimension 3. “Dimension 1” information has to be included in any sentence in order for it to make grammatical sense and thus would naturally feature most prominently and at the basic level in children’s retellings. These items would also be grounded in more obvious visual content like a character’s appearance and the actions they took. “Dimension 1” components therefore would not be as “elaborated” as the inclusion of more optional elements such as the “Dimension 2” components that would require further reflection on or extra processing of the scenes such as temporal and spatial information or how a character actually performed an action. Finally, the most elaborate element of a narrative pertains to the inferential complexity of causality conveyed by “Dimension 3” components.

Throughout the trial-and-error revision process of the coding schema (following Schreier, 2012), we further created a number of more finely grained subcomponents within each main dimension (see Table 3). Note that the selection of the components on the sublevel was derived from/adjusted to the data and thus reflects children’s use of linguistic means to refer to the six main components:

**TABLE 3:** Full details of narrative elaboration coding schema.

**Narrative elaboration coding schema**		
	**Linguistic means**	**Example**
**Component of *Who***		
• Who.1—direct naming of actors, agents or participants • Who.2—indirect references to actors/agents/participants	→ nouns plus articles → gendered pronouns and articles	→ e.g. “the mole” → e.g. “he”, “the (masc.)”
**Component of *What***		
• *What.1*—all general actions including their relevant inanimate objects • *What.2*—actions that highlight the manner of an event • *What.3*—actions that highlight a spatial transition or location	→ general verbs → verbs of manner → verbs with an additionally encoded spatial element	→ e.g. “is”, “went” → e.g. *hüpfen, leuchten* (”hop”, “glow”) → e.g. *hin* + *setzen* (”to place within”)
**Component of *Where***		
• *Where.1*—spatial axis locations • *Where.2*—explicit mentioning of specific story settings/locations	→ spatial prepositions/adverbs → nouns referring to locations	→ e.g. “above”, “inside” → e.g. “the pond”, “the nest”
**Component of *When***		
• *When.1*—temporal sequencing • *When.2*—explicit mentioning of specific temporal locations or points in time	→ temporal prepositions/adverbs → (adjective/adverb +) noun or preposition + noun	→ e.g. “and then”, “before”, “after” → e.g. “last year”, “winter”, “in the night”
**Component of *How***		
• *How.1*—how something looked, felt, sounded, etc. • *How.2*—how something was done	→ adjectives → adverbs of degree and manner, conjunction “with”	→ e.g. “sad” → e.g. “quickly”, “with a shovel [tool]”
**Component of *Why***		
• *Why.1*—weak sequential causality • *Why.2*—stronger inferred causality	→ events listed with an implicit causal sequence → using explicit causal connectives or explicating a purpose/goal	→ e.g. “he couldn’t do it, then he was sad” → e.g. “because” or “so that”, “in order to”

Four further categories of children’s talk: Meta-talk, Associative Talk, Sound Effects and Reported Speech, were created for assigning the remaining communicative resources used by the children and for observational purposes. “Meta-talk” (Schiffrin, 1980, p. 200) referred to any talk or conversation about the process of telling the narrative, e.g. with whom and when they experienced it, or instances of stepping out of the narrative to say something directed at the listener, such as out-of-story comments or signals to their listener for attention or help with constructing the narrative (e.g. yes/no, “hmmm,” “what else…,” “I can’t remember any more,” etc.). “Associative Talk” (Ornstein et al., 2004, p. 382) referred to only the talk from the child that oriented the listener to details in the story using the child’s or shared previous experiences or information (i.e., “the star was green, like that jumper of yours, Mama.”). Any instances where the children quoted dialogue from the characters (e.g. “The hare said: ‘We’ll help you”’.) were coded as Reported Speech, and any instances of onomatopoeia or sound effects (e.g. “Tsching-tsching!” for a shovel hitting a boulder) were coded as Sound Effects. Any repetitions were also coded separately as Repetitions to avoid them exerting any biases on the statistical analysis of the data.

**FIGURE 1:**
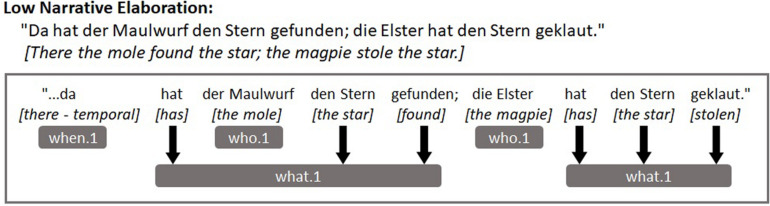
Example of an utterance with a lower level of narrative elaboration and its coding.

**FIGURE 2:**
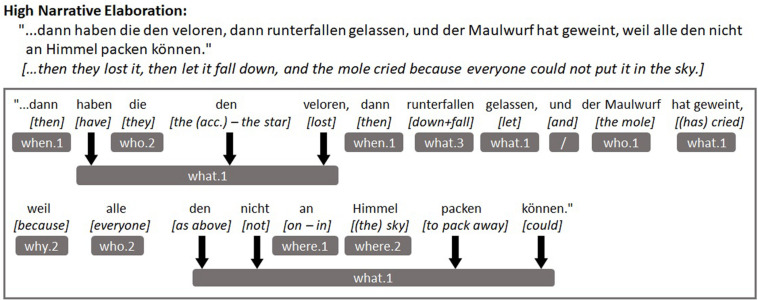
Example of an utterance with high narrative elaboration and its coding.

The Figures 1, 2 below depict example utterances taken from the transcripts of two different children from our sample as well as how these utterances were coded. Figure 1 presents an example of an utterance with a low level of narrative elaboration while Figure 2 shows an example of an utterance with a much higher level of elaboration.

**FIGURE 3:**
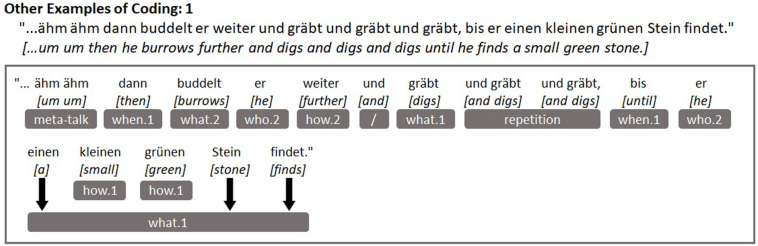
Example utterance demonstrating other coded elements (*what.2, how.1, how.2, meta-talk, repetition*).

**FIGURE 4:**
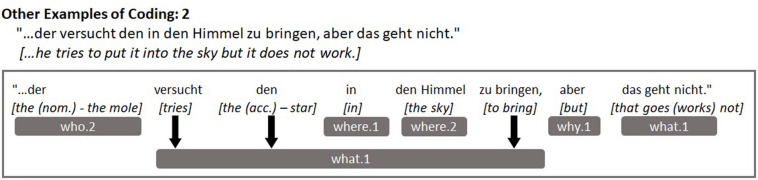
Example utterance demonstrating other coded elements (*why.1*).

**FIGURE 5:**
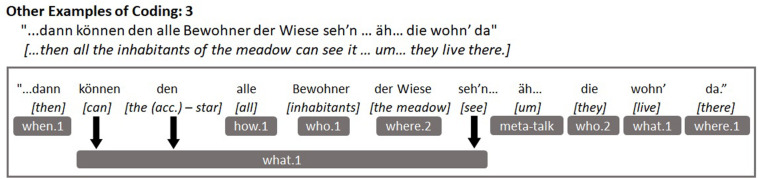
Example utterance demonstrating other coded elements (occasions during which *where.1* and *where.2* can occur separately).

In the interests of replicability, we provide clear examples of children’s utterances from our sample and how they were coded in Figures 3–5 below. Figure 3 demonstrates some of the other narrative components and conversational elements that we coded, which are not shown in Figures 1, 2 above (*what.2, how.1, how.2, meta-talk, repetition*), while Figure 4 depicts an example of the weaker sequential causality or consequences component (*why.1*). Some readers might question whether the spatial components *where.1* and *where.2* could actually appear independently of one another within children’s utterances and Figure 5 illustrates this difference quite effectively. The component *where.2* does not correspond to every noun that could follow a spatial preposition (reflected in *where.1*), rather *where.2* represents mentions of specific locations and settings from a story perspective: Those with contextual narrative importance. So, “in the hand” would only be coded for *where.1* while “through the meadow” would be coded for both *where.1* and *where.2*, because the meadow is a setting in the story in which scenes take place.

Our coding system bridges perspectives on the development of cognitive, linguistic and narrative skills as well as the acquisition of concrete language structures/categories. In this sense, there are also different scopes to story vs. linguistic elaboration. As this is an entirely novel coding schema, questions remain about its validity, including whether the dimensions are related and whether there is any progression from Dimension 1 → Dimension 2 → Dimension 3. Our statistical analysis was designed to assess and respond to these questions and will be discussed further in the Results section. To evaluate coding reliability, 15% of the data was independently coded by two coders. We used Cohen’s kappa to measure inter-rater agreement on the coding schema (κ = 0.609).

## Results

### The Dimensions of Elaboration

All statistical data analysis was conducted using the software IBM SPSS 25 for Windows. Table 4 below shows the children’s average use of each of the dimensions as a proportion of total intonation phrases within the narrative retelling setting as well as the percentage of children who used at least one example of an item coded for each of the dimensions and their (sub)components. All of the children used at least one instance of Dimension 1 and Dimension 2 items, with the group percentages dropping progressively from *Who* to *Why*, although this effect varied more strongly on the sublevel rather than on the main level. Only 58.7% of the children used Dimension 3 items and even then mean use of these items was very low, indicating that this area was more challenging for them. The descriptive results suggest that 4-year-old children first narrate along Dimensions 1 and 2 and progress to 3.

**TABLE 4:** Children’s average use of all narrative elaboration dimensions as a proportion of their total intonation phrases and the percentage of total children (*N* = 46) who used that (sub)component at least once in their narrative.

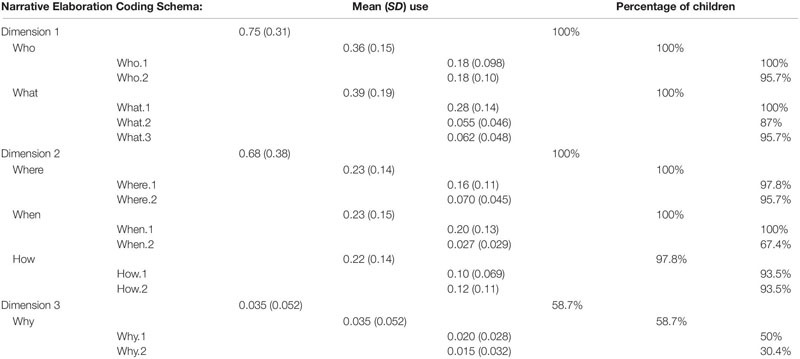		

To explore whether the three dimensions of elaboration are related and thus sum up children’s ability to elaborate, we first conducted Spearman’s correlations between children’s age, their use of the dimensions and their instances of *Meta-talk*. The results are presented in Table 5.

**TABLE 5:** Spearman’s correlations (*N* = 46).

	**Dimension 1**	**Dimension 2**	**Dimension 3**	**Meta-talk**	**Age**
Dimension 1	–	0.78***	0.34*	−0.48**	0.14
Dimension 2	0.78***	–	0.29*	−0.53***	0.13
Dimension 3	0.34*	0.29*	–	–0.070	0.15
Meta-talk	−0.48**	−0.53***	0.070	–	–0.019
Age	0.14	0.13	0.15	–0.019	–

*p < 0.05, **p < 0.01, ***p < 0.001.

The inclusion of Dimension 1 and Dimension 2 components in the children’s narratives were strongly positively correlated with each other, Dimension 2 and Dimension 3 were also significantly positively correlated with one another, as were Dimension 1 and Dimension 3. These results suggest that the dimensions of our coding systems are related and provide support for the idea of one ability being reflected in the three dimensions. There was also a moderate negative correlation between Dimension 1 and *Meta-talk*, as well as between Dimension 2 and *Meta-talk* suggesting that they capture different abilities, but we found no relation between Dimension 3 and *Meta-talk*. No significant correlations were found between the dimensions and the age in months of the children suggesting that the narrative elaboration dimensions are not a matter of children’s age in months.

### Dimensions of Elaboration Under Medial Conditions and in Relation to IQ

In the next part of the analysis, we investigated how the extent of children’s elaboration differed depending upon whether their narrative was a retelling of the video or the book input. Here, we followed the hypothesis that children’s retellings would demonstrate higher narrative elaboration and incorporate more narrative details if they had experienced the original story stimuli in a video format in contrast to a storybook format. In order to conduct our analyses, we combined our hypotheses with those reported in the literature that children’s IQ is related to their performance on narrative retelling tasks (Humphries et al., 2004; Shamir et al., 2018).

We first considered whether the two groups (book vs. video condition) were comparable when it came to their IQ scores. Shapiro-Wilk tests indicated a normal distribution in the wider sample (*n* = 39, *p* = 0.945) as well as in the book (*n* = 23, *p* = 0.865) and video conditions (*n* = 16, *p* = 0.127), so we then conducted an independent samples *t*-test. Our analysis revealed that the two groups differed significantly, *t*(37) = −2.807, *p* < 0.01, with a large effect size of *r* = 0.42 according to Cohen (1992) suggesting that the participants in the video condition (*n* = 16; *M* = 111.88; *SD* = 7.34) had higher IQ scores than the participants in the book condition (*n* = 23; *M* = 103.48; *SD* = 10.26). As a consequence of this significant difference, we conducted analyses of covariance (ANCOVAs) for further group comparisons to assess the effect of the medial conditions on narrative (sub-)components in retellings under consideration of the children’s IQ scores. The inclusion of children’s IQ scores as a covariate results in a corrected model.

Including children’s IQ as a covariate (ANCOVA) when comparing the groups (those who received book vs. video input) across Dimension 1 (Table 6), we found a moderate effect suggesting that proportions of use were higher in the video condition (*n* = 16; *M* = 0.90; *SD* = 0.41) than the book condition (*n* = 23; *M* = 0.63; *SD* = 0.22), *F*(1, 37), *p* = 0.03, *eta*^2^ = 0.13. To reveal what content was narrated differently, we further conducted additional ANCOVAs at the sublevel and found that both the *Who* and *What* components differed significantly between conditions, with the video group outperforming the book group in each of them. When the next sublevel was considered, these significant between-condition effects appeared to be driven by children’s *What.1* and *Who.2* use (see Table 6).

**TABLE 6:** Dimension 1 (D1) differences between conditions with Mean (Standard Deviation), *n* = 39.

	**D1**	**What**	**What.1**	**What.2**	**What.3**	**Who**	**Who.1**	**Who.2**
Book *n* = 23	0.63 (0.22)	0.32 (0.12)	0.23 (0.09)	0.04 (0.04)	0.05 (0.04)	0.31 (0.13)	0.18 (0.12)	0.14 (0.07)
Video n = 16	0.90 (0.41)	0.48 (0.25)	0.32 (0.19)	0.07 (0.05)	0.08 (0.06)	0.42 (0.18)	0.18 (0.09)	0.25 (0.12)
**ANCOVA with IQ as covariate**								
*p*	0.03*	0.04*	0.01*	0.06	0.12	0.04*	0.90	0.003**
*F*	5.30	4.79	2.91	3.66	2.55	4.37	0.01	10.54
*eta*^2^	0.13	0.12	0.08	0.09	0.07	0.11	0.01	0.28

*p < 0.05, **p < 0.01, ***p < 0.001.

Conducting an ANCOVA to investigate group differences across children’s Dimension 2 usage, we found a moderate effect, according to which, again, the proportions of use were significantly higher in the video condition (*n* = 16; *M* = 0.83; *SD* = 0.49) than the book condition (*n* = 23; *M* = 0.53; *SD* = 0.24), *F*(1, 37), *p* < 0.05, *eta*^2^ = 0.11. Further ANCOVAs on the sublevel (see Table 7) revealed that this effect appeared to be driven predominantly by the *Where.2* subcomponent.

**TABLE 7:** Dimension 2 (D2) differences between conditions with *M* (*SD*), *n* = 39.

	**D2**	**Where**	**Where.1**	**Where.2**	**When**	**When.1**	**When.2**	**How**	**How.1**	**How.2**
Book *n* = 23	0.53 (0.24)	0.18 (0.11)	0.13 (0.09)	0.05 (0.03)	0.17 (0.10)	0.15 (0.08)	0.02 (0.03)	0.18 (0.10)	0.10 (0.07)	0.09 (0.06)
Video n = 16	0.83 (0.49)	0.28 (0.17)	0.20 (0.13)	0.09 (0.05)	0.28 (0.18)	0.24 (0.15)	0.04 (0.03)	0.27 (0.18)	0.10 (0.06)	0.17 (0.16)
**ANCOVA with IQ as covariate**										
*p*	0.048*	0.07	0.12	0.04*	0.08	0.08	0.26	0.10	0.57	0.08
*F*	4.20	3.57	2.51	4.49	3.31	3.36	1.27	2.84	0.33	3.20
*eta*^2^	0.11	0.09	0.07	0.11	0.08	0.09	0.03	0.07	0.01	0.08

*p < 0.05, **p < 0.01, ***p < 0.001.

Regarding the Dimension 3 *Why* component and other coded conversational elements (Meta-talk, Associative Talk, Sound Effects, and Reported Speech), no significant effects of condition were found suggesting that the beneficial effects of the video condition pertain to Dimensions 1 and 2.

In summary, after applying ANCOVAs which took children’s IQ scores into account as a covariate, we found moderate to large significant effects of medial condition on children’s proportion of use of the narrative components *Who*, *What* and *Where*, reflected predominantly in their use of the *Who.2*, *What.1*, and *Where.2* subcomponents. For each of these (sub-)components, children from the video condition used them more frequently than the children from the book condition.

## Discussion

The emergence of new digital media and technologies is dynamically influencing how children interact with other people, objects, and the world around them. Previous studies have found that narrative input conveyed through diverse media influences the structure and content of children’s narrative retellings as well as differentially affecting their word learning and story comprehension. Visual information conveyed by televised (but not storybook) narratives may be easier for kindergarten/pre-school children to process than verbal or language-based input, promoting greater story comprehension, supporting mental encoding processes and facilitating a more detailed event recall (Beck and Clarke-Stewart, 1998; Linebarger and Piotrowski, 2009). The mode of presenting information may play a further role, with bimodal (visual and verbal) presentation and audiovisuals that convey more obvious emotional content having additive effects on memory (Beck and Clarke-Stewart, 1998). Narratives presented in a televised format or in the form of a digital storybook with visual film-like enhancements appear to boost story knowledge and elicit richer and more detailed narrative retellings than traditional, orally transmitted storybook media, which have been shown to better promote word learning and character references (Beentjes and van der Voort, 1991a,b; Podszebka et al., 1998; Linebarger and Piotrowski, 2009; Sarı et al., 2019; Diehm et al., 2020). These findings all suggest that the linguistic content and plot components of children’s narratives might be differentially affected by the medium of input to which they are exposed. Since narrative content has to be construed, a great challenge for children is to make inferences about and remember the original story’s plot. This cognitive effort is reflected in studies indicating that children’s narrative retelling is related to individual differences in IQ and (socio-)cognitive development (Humphries et al., 2004; Genereux and McKeough, 2007; Nicolopoulou and Richner, 2007; Dore et al., 2018; Shamir et al., 2018). Our study attempted to address this issue, evaluating the linguistically encoded story components of narrative retellings as a function of differing media inputs, with additional consideration of children’s IQ as an influential variable.

In our study, 46 typically developing children from Germany, aged 4–5 years, participated. They were randomly assigned to one of two medial conditions (watching a DVD vs. reading a storybook). They were assessed during two sessions: One, in which they retold a story to a different caregiver than the person with whom they had experienced the stimuli story (from either watching a DVD or joint reading of a storybook); and two, in which the children’s IQ was assessed by conducting the SON-R test (Tellegen et al., 2007). The children’s retellings were coded using a specifically developed coding system that captures their use of narrative elaboration and its different dimensions. Our analyses compared children’s retellings between the medial conditions by taking their IQ scores into consideration as a covariate. Our results showed that the children from the video condition gave significantly more elaborated retellings, particularly across various *Who*, *What*, and *Where* components on the sublevel, whereas differences between the medial conditions in the components *When*, *How*, and *Why* did not reach statistical significance. Given the findings of previous studies, we did expect that the narrative retellings of the children from the video condition would be more detailed than those from the book condition and our results are consistent with this line of research. Despite having this expectation, it is still striking to find that such differences exist, since the children in the book condition had access to rich verbal input and linguistic information while children in the video condition only experienced non-verbal visual input with minor background sound effects. This could have easily primed the children from the book condition for success in the retelling task by pre-furnishing them with the necessary vocabulary and grammatical structures, but the children from the video condition still appeared to outperform them. It is possible then that for children at this age, the advantages of visually conveyed information supersede that of audially conveyed information.

Examining the dimensions’ particular components more closely, we found that information from the sublevel about *What*, *Who*, and *Where* were more frequently incorporated within the retellings of the children from the video condition than the book condition, but differences between the conditions for *When*, *How*, and *Why* did not reach statistical significance. To distinguish more finely between the different language and story content narrated by the children, we also analysed the children’s use of the sublevels of the *What*, *Who*, *Where*, *When*, *How*, and *Why* components.

In the video condition, children used more Dimension 1 components as a whole than children from the book condition but there were also specific differences. On the sublevel, *What* was separated into: (1) general verbs and actions, (2) verbs of manner (e.g. shines, hops), and (3) (German) verbs that encode an additional spatial element (e.g. climbs high). We found significantly higher use of the first component in the video condition. Since this subcomponent is encoded in children’s use of more general or basic verbs and these are often syntactically necessary for an utterance, it is possible that frequency of use of this subcomponent may reflect frequency of narrative detail inclusion. Equally, it might also be a language-specific effect of syntax. From our findings concerning the *What* component, it is likely that experiencing visually transmitted information about the actions that constitute a story (literally seeing them happen) benefits encoding of actions in general and resulted in a stronger memory trace for children from the video condition in contrast to the children in the book condition who only heard about these actions occurring.

*Who* was separated into: (1) direct references to the characters in the story (e.g. “the mole”) and (2) indirect references to characters (e.g. “he,” “they,” etc.). In German, the possibilities for using indirect references are more extensive than in English, as the genders of nouns allow for the use of only the article to distinguish characters. In our study, we only found significant effects between the conditions regarding the use of the second subcomponent (*Who.2*). Use of the *Who.1* subcomponent was not different. However, as indicated by the large effect size, the children in the video condition used more *Who.2* (indirect character reference) items than those in the book condition. It is possible that having experienced the visual input from the video, those children may have been better supported in their mental encoding of the characters within the story and consequently mentioned these characters more frequently, providing more information about them in their retellings. Having a potentially stronger memory trace of the characters from which to construe their retelling might have led to the children feeling less need to directly name them for their audience. It might be the case that this subcomponent better reflects children’s performance on the *Who* component in general, as it would be syntactically and pragmatically unnatural to constantly refer to the full name of the character (encoded by *Who.1*). However, there are also quite a large number of characters in the stimuli story so it could still pragmatically make sense to refer to the main character by fully naming them regularly. Since the video was non-verbally presented, it may be the case that children from the book-condition received more input directly naming the character nouns than those children from the video condition.

Regarding the Dimension 2 components (*Where*, *When*, and *How*), the participants from the video condition used more of these components on the whole but here too there were specific differences at the level of the individual (sub-)components. After taking the children’s differences in IQ into consideration, only an intermediate effect concerning the second subcomponent of *Where* (*Where.2*) was found to be statistically significant. The two subcomponents of *Where* represented: (1) spatial location prepositions and directions (e.g. up, in, above) and (2) explicit mentioning of specific story-related setting locations (e.g. the nest, the cave, the forest). Children in the video condition used the second subcomponent significantly more in their retellings than those in the book condition. It is likely that the specific story setting locations involved in the story were more obviously conveyed to the children in the video condition through the visual format, influencing their memories of the scenes. This effect could also have been continuously reinforced by their witnessing of the scenes taking place within these specific story settings/background locations (as well as the transitions between them) in contrast to the children in the book condition who would have had to construct their own mental representation of the scene on the basis of audial descriptions mentioned in the book only at the beginning of the narrative and therefore occurring less frequently. It might also be the case that the video facilitated stronger mental encoding of the spatial axis locations but that these were also frequently explicitly named, i.e., linguistically encoded by parents during the oral reading of the book, allowing them to perform in a manner similar to children from the video condition.

The subcomponents of *When* were divided along similar principles to *Where* into: (1) sequential aspects of the story (e.g. before, then, after) and (2) explicit mentioning of specific points in time (e.g. in the spring, at night). After children’s IQ had been taken into account, neither of these subcomponents were found to be significantly different when the conditions were compared. If children used the first subcomponent (*When.1*) more frequently, it may be simply that they remembered more information about the story as a whole and thus included more instances of sequencing of these story elements. Since children acquire the temporal sequencing aspect of narrative structure very early on, it is possible that no significant differences were found between the conditions because they were already developmentally past this point. The second subcomponent (*When.2*) was the least frequently used of all of the Dimension 1 and 2 (sub-)components (see Tables 4, 6, 7) by children across the sample, so that it is possible that we simply did not have enough instances in the data to appropriately evaluate this component. Clearly, further research is needed in larger samples matched for IQ with targeted stimuli to systematically manipulate and better evaluate children’s use of this component.

The component *How* was separated into: (1) how something looked or felt (adjectives), and (2) how something was done (adverbs and with what tools). Neither subcomponent reached statistical significance once the children’s IQ differences were considered. With regard to the lack of findings concerning the first subcomponent (*How.1*), it is possible that the adjectives most relevant to the particular story stimuli used in our study (e.g. the colour of a particular star and the size of the mole) were both integral or explicit enough information to the story in both conditions. According to this explanation, we simply had very few differences between the children’s retellings. This first subcomponent also included information about characters’ feelings or traits (e.g. sad, happy) which might have been equally or even more explicit in the book condition (as discussed above regarding the previous work on character references: Beentjes and van der Voort, 1991a,b; Podszebka et al., 1998). This means that there could have been confounding effects within this first subcomponent because of the way in which we grouped adjectives in our coding schema. On the one hand, the visual input may have made the visual characteristics of adjectives more explicit but the relevant emotional adjectives less so, while the audial storybook input might have done the opposite. Further research may need to narrow down the types of adjectives used by the children in order to find more fine-grained differences between the conditions. Theoretically, the second subcomponent (adverbs conveying how something was done or extra information about the tools used) could have potentially led to between-condition differences because of the inherently visual elements that adverbs often convey about an action: additional information about its degree, speed or visual features, which might have been visually reinforced for the children in the video condition. It is difficult to pinpoint the reason for a lack of an effect in this subcomponent. It would require more fine-grained research on the individual participant level but children in the book condition may have performed similarly as a result of experiencing reinforced linguistic encoding of these elements. It is also possible that individual differences in IQ, access to linguistic means, and cognitive performance could offer potential avenues to pursue this topic further.

The Dimension 3 component *Why* was separated into: (1) weak sequential causality and consequence (*Why.1*: something happened, then a related other thing happened) and (2) strong inferred causality (*Why.2*: children would have to infer and encode something about the character mental reasons for doing something). Between medial condition, neither of these subcomponents was different. The lack of difference in children’s use of *Why* between conditions might be due to the inherent cognitive complexity in construing causality (Makdissi et al., 2019). As the children in our study were only between 4 and 5 years old, it may be that they are not yet at a point in their development where they can cognitively and consistently cope with more complex questions about the *whys* (explicit causality). Only half of all participants used at least one instance of weak sequential causality and just under a third used a least one instance of the stronger inferred causality. The *Why* (sub-)components were not significantly correlated with the children’s IQ scores in our sample. Previous research has found that incorporation of these elements (explanatory and interpretative clauses about character motivations and causality) within narrative retellings is related to children’s age and sociocognitive development in later childhood (Genereux and McKeough, 2007; Nicolopoulou and Richner, 2007; Colletta et al., 2010; Pavias et al., 2016; Hamilton et al., 2020). In order to talk about the *whys* of the story presented to them, children are required to both comprehend and infer a number of implicit story details and then construe them mentally and linguistically to make them accessible to their narrative audience. It is possible that if the study were to be repeated with older children, that those in a book condition might be more effective at incorporating elements of causality and underlying character motives, as written narratives often present these more explicitly than visual narratives. Prior research has found increased levels of such character references in those reading storybooks rather than watching televised narratives (Beentjes and van der Voort, 1991a,b; Podszebka et al., 1998). Cognitive and affective perspective-taking may also be tapped differently by media of input: a video might place the child into the role of a more distanced observer required to infer many implicit details about the characters’ motives while a book offers a more direct, explicit and involved insight into a character’s mind.

Taken together, the findings presented here indicate that under the consideration of children’s individual differences in IQ scores different medial condition entail variation in narrative retellings. The current paper extends the work of previous authors by pinpointing differences in the specific story components that children include in their narrative retellings after watching a non-verbal video versus being read a traditional storybook. Together with previous research, we propose that the differences stem from memories that are specific to a medium (video or book). Overall, children who had experienced the video input were better supported and included a greater proportion of distinct narrative details in their retellings than those who had been read the storybook. Our study was also particularly interesting because the caregivers involved had no prior experience of the story being retold to them and were not “knowing co-tellers,” so the children had epistemic primacy in this situation and ownership of the story (Takagi, 2019, p. 107) allowing us to hone in authentically on their individual narrative skills.

## Limitations

We are aware of some limitations of our study: Firstly, our sample sizes were unbalanced with regard to several aspects. This was unfortunately due to the recruitment and random assignment of participants as well as the emergent issues discussed in the methods section leading to the data of some children having to be excluded from the analysis. As a result, we had fewer participants for the medial condition analyses (*n* = 39) than the wider narrative elaboration coding schema analyses (*N* = 46). For the narrative elaboration analyses which examined the coding schema in general, we had 4 fewer participants in the video condition (*n* = 21; 10 male) than the book condition (*n* = 25; 17 male). Although the genders were fairly balanced in the video-condition group, there were many more boys than girls in the storybook condition. Due to unforeseen circumstances preventing the collection of all the IQ data, for the medial condition analyses, we had 7 fewer participants in the video condition (*n* = 16; 9 male) than in the book condition (*n* = 23; 16 male). Again, the genders were fairly balanced in the video condition but there were many more boys than girls in the story condition. Clearly, thus, the comparisons between the conditions need to be interpreted with caution, also because in our sample and by random assignment, children from the video condition did differ in their IQ scores from children of the other condition significantly. Future research with larger groups could consider more fine-grained analytical approaches with the assignment of participants to higher and lower IQ groups in order to investigate differences in narrative elaboration on a more individual level.

Secondly, the storybook that we used did also have some supporting illustrated pictures scattered throughout it. This may have aided the children in the book condition in a similar way to the visuals presented in the video condition. Despite this potential issue that could have weakened the effects between the conditions, our results still revealed significant (moderate to large) differences between conditions.

Finally, our study concentrated on the linguistically encoded story content of narrative retellings; for future research, it might be informative to examine potential differences between the conditions in the structure of the retellings, as the children experiencing storybook narratives are exposed to an arguably more explicitly linguistically schematised structure (e.g. “There was once a …”, “then one day … happened”, “later that day …”, “the end.”, etc.) than those watching televised narratives who have to infer these details. Further longitudinal studies might also focus on how the dimensions of our coding schema match levels of increasing competence.

## Implications and Future Opportunities

Our findings line up with previous research and indicate that today’s digital technologies can offer a positive environment for children’s development, education, and their interaction with the world around them. The results of this study have ramifications within three main areas: (1) child development, (2) education, and (3) further research.

Regarding children’s development and education: If exposure to visual input supports children’s comprehension and encoding of information as well as their subsequent retelling of that information, then these formats could be utilised to scaffold children’s learning and development. While our results only confirm the advantages of visual media input on a few narrative components once the IQ is taken into account, at the very least no disadvantages could be found. This means that children might learn storytelling from movies just as well as they do from books, at least regarding the content-based components under investigation within this paper. In all aspects of life, children learn from their experience of the world around them. Information conveyed in a visual format is crucial to this process and storytelling may be no different. For this reason, it is possible that children may gain much more from simply viewing media content than might be initially anticipated. Perhaps experiencing a more visual source of input frees up the cognitive resources needed to best process and encode that information (Goldin-Meadow et al., 2009). Educators could thus consider developing a dual system that takes advantage of the opportunities available to get children actively engaging with and building upon information or media content that has first been presented to them through a more visual means. The benefits of visual input might even extend to children’s comprehension of oral storytelling when supported with iconic gestures. If this is the case, then discourse, parenting, and teaching techniques that make greater use of visual supports (both manual and digital) could be developed for use at home, in school and in intervention settings.

While filling some gaps, our study has also identified new directions and opportunities for research in our field. Future work should take a more finely grained approach to investigate which kinds of stories and language are best supported by which format and how these formats can be most effectively deployed to individually scaffold children’s development. Future studies could identify and systematically manipulate the presentation of each narrative component (the *Who*, *What*, *Where*, *When*, *How*, and *Whys* of a story) to explore how their encoding and retelling might be best supported through visual input and how it resonates with children who score differently in an IQ test. Other work could also examine the differences in the event structuring of children’s narrative retellings and explore how each media format differs in promoting this aspect of their narrative skill development. Traditional storybooks may provide interaction training with a modelled structure whereas video input might require the child to construct a narrative retelling more independently. It would also be interesting to explore multimodality in children’s retellings and the function of their gestures that accompany their verbal behaviour: Differences between the conditions in the use of plot components might also be reflected in their use of gestures and gestural viewpoints. Combining gesture and language analysis might then tell us more about children’s underlying representations of the narrative events. Finally, there is the question of research design and the selection of stimuli for narrative retelling tasks: If narrative elaboration is better supported by visually conveyed input, then researchers have an ethical responsibility to take this into consideration when designing their experiments and interpreting their data.

## Data Availability Statement

The datasets presented in this article are not readily available because: the datasets generated for this study cannot be made publicly available because participants did not consent to future re-use of their data by other researchers. Requests to access the datasets should be directed to Camilla Crawshaw, camilla.crawshaw@tu-dortmund.de.

## Ethics Statement

The studies involving human participants were reviewed and approved by the Ethical Committee of the Bielefeld University (EUB 2014-111). Written informed consent to participate in this study was provided by the participants’ legal guardian/next of kin and the children were given the opportunity to withdraw from the experimental interaction at any time.

## Author Contributions

KR and FK contributed to the conception, design, and piloting of the wider study. FK, KR, and UM recruited participants and conducted data collection. KR and CC developed the coding schema. UM and FK contributed to discussions about the coding schema. CC and UM coded the data. CC, KR, and UM conducted data analysis. CC, FK, KR, and UM drafted the manuscript. All authors commented on, edited, and revised the manuscript prior to submission.

## Conflict of Interest

The authors declare that the research was conducted in the absence of any commercial or financial relationships that could be construed as a potential conflict of interest.
